# Antifungal activity of Allyl isothiocyanate by targeting signal transduction pathway, ergosterol biosynthesis, and cell cycle in *Candida albicans*

**DOI:** 10.22034/CMM.2023.345081.1429

**Published:** 2023-06

**Authors:** Shivani Balasaheb Patil, Ashwini Khanderao Jadhav, Rakesh Kumar Sharma, Sargun Tushar Basrani, Tanjila Chandsaheb Gavandi, Sayali Ashok Chougule, Shivanand Ramappa Yankanchi, Sankunny Mohan Karuppayil

**Affiliations:** 1 Patil Education Society (Deemed to be University), Kolhapur-416-006, Maharashtra, India; 2 Department of Stem Cell and Regenerative Medicine and Medical Biotechnology Centre for Interdisciplinary Research, Maharashtra, India; 3 Patil Medical College Hospital and Research Institute, Kadamwadi-416012-, Kolhapur, Maharashtra, India; 4 Department of Zoology, Shivaji University, Kolhapur- 416004, Maharashtra, India

**Keywords:** Allyl isothiocyanate, *Candida albicans*, Ergosterol biosynthesis, RT-PCR, Silkworm

## Abstract

**Background and Purpose:**

In recent years, the inclusion of *Candida albicans* on the list of infections that pose a threat due to drug resistance has urged researchers to look into cutting-edge and effective antifungal medications. In this regard, the current study investigated the probable mode of action of allyl isothiocyanate (AITC) against *Candida albicans*.

**Materials and Methods:**

In this study, planktonic assay, germ tube inhibition assay, adhesion, and biofilm formation assay were performed to check the growth and virulence factors. Furthermore, ergosterol assay, reactive oxygen production analysis, cell cycle analysis, and quantitative real-time polymerase chain reaction analysis were performed with the aim of finding the mode of action. A biomedical model organism, like a silkworm, was used in an *in vivo* study to demonstrate AITC anti-infective ability against *C. albicans* infection.

**Results:**

Allyl isothiocyanate completely inhibited ergosterol biosynthesis in *C. albicans* at 0.125 mg/ml. Allyl isothiocyanate produces reactive oxygen species in both planktonic and biofilm cells of *C. albicans*. At 0.125 mg/ml concentration, AITC arrested cells at the G2/M phase of the cell cycle, which may induce apoptosis in *C. albicans*. In quantitative real-time polymerase chain reaction analysis, it was found that AITC inhibited virulence factors, like germ tube formation, at 0.125 mg/ml concentration by downregulation of *PDE2*, *CEK1*, *TEC1* by 2.54-, 1.91-, and 1.04-fold change, respectively, and upregulation of *MIG1*, *NRG1*, and *TUP1* by 9.22-, 3.35-, and 7.80-fold change, respectively. The *in vivo* study showed that AITC treatment successfully protected silkworms against *C. albicans* infections and increased their survival rate by preventing internal colonization by *C. albicans*.

**Conclusion:**

*In vitro* and *in vivo* studies revealed that AITC can be an alternative therapeutic option for the treatment of *C. albicans* infection.

## Introduction

In the past few years, incidence rates of fungal infections have increased tremendously. Annually, around 10 lakh deaths occur due to fungal infections across the world. The fungal infection mainly occurs in immunocompromised individuals [ 1 , 2 ]. Among these, fungal infections caused by *Candida albicans* are the most common threat to human beings [ 3 , 4 ]. *Candida albicans* is a pleiomorphic fungal pathogen that has the capacity to produce biofilms on the cell surfaces of mammals as well as implanted medical devices [ 5 - 7 ].

It has the ability to form biofilm on both biotic and abiotic surfaces, like central venous system catheters, urinary catheters, stents, porcine heart valves, artificial heart valves, intrauterine devices, and artificial knee caps. The colonized prosthetics may act as a permanent source of bloodstream infections. The majority of the studies suggest that biofilm-associated infections in patients are difficult to eradicate as biofilms are resistant to standard antifungals [ 8 ]. Therefore, the treatment of biofilm-related infections has become a major challenge to clinicians [ 9 ]. Due to drug tolerance, it may be necessary to increase the dosages of the drugs beyond the therapeutic range. This is not always advisable due to the increased side effects of the drugs. 

Since the biofilms are hard to eradicate, the only available alternative may be the physical removal of the devices. In addition, colonized devices, such as pacemakers, may malfunction. Clogging of the catheters by *C. albicans* is a serious problem, especially in diabetic or immuno-compromised persons since the catheters require replacement. Removal or replacement of prosthetics is expensive and uncomfortable for the patients and may require frequent visits to the doctor as well as hospitals contributing to the additional expenses, mortality, and morbidity [ 10 ]. 

This necessitates an alternative treatment modality to counter the low efficacy, significant side effects, and emergence of multidrug-resistant *C. albicans* strains. Biofilms have the ability to adhere and accumulate on numerous surfaces [ 6 ]. The new antifungal drug strategy should concentrate on the development of drugs that prevent and remove biofilms. Numerous plant-derived substances have been shown to possess potential anti-*Candida* activities through a variety of mechanisms, including inhibition of the yeast-to-hyphae transition, prevention of the formation of biofilms, impairment of cell metabolism, cell wall integrity, cell membrane fluidity, and apoptosis [ 11 ].

Allyl isothiocyanate (AITC) is a natural compound derived from cruciferous vegetables. An earlier study performed by Raut et al. [ 12 ] has suggested that AITC alone and in combination with the standard antifungal, fluconazole (FLC), successfully inhibits the growth and virulence factors of *C. albicans*. Along with this, AITC at its planktonic and biofilm inhibitory concentration was non-hemolytic in nature. Hence, it can be used as an alternative therapeutic option for the treatment of candidiasis. The present study aimed to verify the mode of action of AITC against *C. albicans*. 

In this study, AITC was explored to better understand its activity and modes of action against *C. albicans*. In this regard, the antifungal properties of AITC were demonstrated in planktonic and biofilm forms of *C. albicans*. Furthermore, AITC exhibited anti-C. albicans efficacy via a variety of mechanisms, like the inhibition of ergosterol production, the effect of AITC on signal transduction gene involved in yeast to hyphal morphogenesis, cell cycle arrest, and reactive oxygen species (ROS) production. These discoveries collectively shed light on the modes of action of AITC against *C. albicans*.

## Materials and Methods

### Fungal strain, growth conditions, and Chemicals

In the present study, *C. albicans* strain ATCC 90028 was used, which was obtained from the Institute of Microbial Technology (IMTECH), Chandigarh, India. The culture was maintained on Yeast Extract Peptone Dextrose (YPD) Agar Plates and slants and maintained at 4°C. *Candida albicans* cells were grown in YPD broth and incubated at 30°C on an incubator shaker at 120 rpm for 24 h. After 24 h, the cells were collected by centrifugation, and the cell pellet was washed with phosphate buffer saline (PBS) twice and used for varieties of assays in the current study. Fetal bovine serum (FBS) was used for yeast to hyphal (Y-H) form transition of *C. albicans*. The RPMI1640 (w/L- Glutamine w/o sodium bicarbonate) medium was used for micro broth dilution assay to determine minimum inhibitory concentrations (MICs) against planktonic and biofilm growth.

The AITC was purchased from Sigma Aldrich Chemical Ltd., Mumbai, India. It was dissolved in dimethyl sulfoxide (DMSO) to make a stock solution of 2 mg/ml. This stock was further diluted and used for *in vitro* and *in vivo* experiments in the current study against *C. albicans*. Moreover, FLC was used as a standard antifungal agent in the current study against *C. albicans*. Experiments were performed according to the guidelines of Clinical Laboratory Standards Institutes (CLSI) micro broth dilution method to determine MICs [ 13 ].

### Determination of minimum inhibitory concentration for Candida albicans planktonic growth

According to the guideline of CLSI M27-Ed4 [ 14 ], the effect of AITC alone and in combination with FLC on the planktonic cells of *C. albicans* was assessed with the help of micro broth dilution method in a 96-well microtiter plate [ 13 ]. Various concentrations of AITC ranging from 0.0039 to 2 mg/ml and FLC from 0.097 to 50 µg/ml were prepared in RPMI-1640 and added to the 96-well plate. Cells without test molecules were considered the control cells. The plates were incubated for 48 h at 35 °C. To examine the cell density, absorbance was taken at 620 nm using a microtiter plate reader (Multiskan Ex, Thermo Electron Corp., USA). The concentration of AITC which caused a 50% reduction in growth, compared to the control, was considered the MIC_50_ for the growth of *C. albicans* [ 13 , 15 ].

### Determination of minimum fungicidal concentration

Effect of AITC and FLC on the growth of *C. albicans* was visibly assessed using a minimum fungicidal concentration (MFC) assay after performing MIC. In total, 10 µl of cell suspension from the wells of MIC_50_ and above MIC_50_ concentrations of AITC and FLC were selected to assess MFC and spread on the YPD agar plate. The YPD agar plates were incubated at 30 °C for 48 h. After 48 h, the plates were observed. The ones that showed less growth or no growth were selected and photographed [ 15 ].

### Estimation of Ergosterol Content

In total, 50 ml of sabouraud dextrose broth was inoculated with a single colony of *C. albicans* from a sabouraud dextrose agar plate. Planktonic MIC_50_ and sub-MIC concentrations of AITC within the range of 0.0078 to 0.125 mg/ml were added into different flasks, without AITC treatment served as control. It should be mentioned that flasks were incubated for 16 h. *Candida albicans* cells were harvested by centrifugation at 2,700 rpm (856 g) for 5 min. The net weight of the cell pellet was determined. Each pellet was mixed with 3 ml of a 25% alcoholic potassium hydroxide solution and was vortexed for 1 min. The cell suspension was transferred to sterile borosilicate glass screw-cap tubes and incubated in an 85 °C water bath for 1 h. The tubes were left to cool after the incubation. 

The next step was to extract the sterols by the addition of 3 ml of n-heptane and 1 ml of sterile distilled water, and vortexing for 3 min. The heptane layer was put into a sterile screw-cap borosilicate glass tube and kept at 20 °C. In order to conduct the analysis, a 0.6 ml aliquot of sterol extract was multiplied five times in 100% ethanol before being spectrophotometrically scanned between 240 and 300 nm. Due to the presence of ergosterol, the isolated material shows a recognizable four-peaked curve. A flat line demonstrated the absence of ergosterol in the extracts [ 16 ].

### Measurement of reactive oxygen species production for planktonic and biofilm cells of Candida albicans

Using 2′, 7′ - dichlorofluorescein diacetate (DCFH-DA), *C. albicans* cells were assessed to find the production of ROS. Cells were harvested by centrifugation, subjected to planktonic MIC_50_ concentration of AITC and 1.5 mM/L of hydrogen peroxide for 4 h, and then washed once with PBS before being re-suspended in 0.5 mL PBS. Afterward, they were incubated at 30 °C for 30 min in the dark followed by the addition of 10µM DCFH-DA. 

The developing biofilm of AITC-treated (0.5 mg/ml) and non-treated *C. albicans* was formed in a 12-well treated microtiter plate. After 24 h, the wells were washed with PBS and exposed to 10 µM DCFH-DA. Following incubation for 1 h at 37 °C, the biofilms were washed with PBS.

A spectrofluorometer FP-8300 (Jasco) was used to determine the fluorescence intensities of re-suspended planktonic and biofilm cells of *C. albicans* [ 6 , 17 ].

### Cell cycle analysis

Log phase *C. albicans* cells were treated with AITC in RPMI medium for about 6 h at 30 °C and then washed twice with chilled PBS (pH 7.0). After washing, the cells were fixed overnight in 70% chilled ethanol at 4 °C. The next day, the cells were washed with PBS and incubated with 10 μg RNaseA, followed by the addition of 50 μg/ml propidium iodide. After 30 min of incubation at 4°C, the cells were analyzed using FACS (FACS Diva Version 6.1.3) [ 18 ]. 

### Germ tube formation inhibitory assay

The FBS growth media was used to assess the effect of AITC on the induction of the germ tube of *C. albicans*. In control and test wells of the 96-well plate, 1×10^6^ cells/ml were inoculated and concentrations of AITC ranging from 0.0039 to 2 mg/ml and FLC from 0.097 to 50 µg/ml were prepared in 20% serum and added to a plate. The plates were incubated at 37 °C for 2 h at 200 rpm. The formation of germ tubes by the cells was observed by using Inverted Microscope. The number of yeast cells and hyphae were counted with the help of Microscope [ 5 ].

### Scanning Electron Microscopy

Germ tube formation of *C. albicans* cells was allowed to form on Foley’s catheter. The MIC_50_ concentration of germ tube formation of AITC and cells (1×10^6^ cells/ml) was added to a 12-well plate as a test and the cells without AITC in the plate served as the control. Both test and control cells were incubated at 37 °C and 50 rpm for 90 min. The samples were fixed in 2.5% glutaraldehyde in 0.1 M phosphate buffer (pH 7.2) for 24 h at 4 °C for scanning electron microscopy (SEM) preparation. The samples were dehydrated in a succession of grades of alcohols after being post-fixed for 4 h in an osmium tetroxide 2% aqueous solution. Samples were mounted on stubs and coated with gold using an automated gold coater. A scanning electron microscope was used to capture images [ 19 ].

### Adhesion assay

To assess the effect of AITC on the adhesion of *C. albicans*, a polystyrene tissue culture 96-well plate was used. The AITC in a concentration range of 0.0039 to 2 mg/ml and FLC from 0.097 to 50 µg/ml were prepared in PBS and added to the well plate along with 1×10^7^ cells/ml. The plate was incubated for 90 min at 37 °C on an orbital shaker with 100 rpm. After incubation, the wells were washed with PBS to remove non-adhered cells. The density of the adherence of cells in each well was examined by relative metabolic activity using XTT assay. The concentration at which a 50% reduction was seen, compared to the control was considered the MIC concentration [ 3 ]. 

### Biofilm assay

### Development of biofilm and mature biofilm

To assess the effect of AITC and FLC on the developing and preformed biofilm, *C. albicans* biofilm was prepared in a 96-well polystyrene tissue culture plate. A range of AITC concentration from 0.0039 to 2 mg/ml and FLC from 0.097 to 50 µg/ml was prepared in RPMI-1640 medium and added along with 1×10^7^ cells/ml into a 96-well plate. The plate was incubated for 48 h at 37 °C. After incubation, the wells were washed with PBS and XTT metabolic assay was performed to analyze biofilm growth. *Candida albicans* 1×10^7^ cells/ml were allowed to mature for 24 h at 37 °C. After incubation, the wells were washed with PBS, and drug dilution prepared in RPMI-1640 medium was added to the plate and incubated for 48 h at 37°C. Finally, the biofilm was analyzed using XTT metabolic assay [ 20 ]. 

### XTT assay for quantification of biofilm

Growth of the biofilm was measured using the XTT metabolic test. The wells containing biofilms were filled with PBS to remove the non-adhered cells and then incubated with 100 μl of XTT-Menadione solution in the dark at 37 °C for 5 h. Color formation by the water-soluble formazan product was measured at 450 nm using a microplate reader [ 20 ].

### Toxicity assay

Human red blood cells (RBCs) were used to study the toxicity of AITC. There were no ethical concerns involved in the toxicity assay process. The institutional Ethical Committee gave its approval to the toxicity assay protocol. Centrifugation was performed on the blood (5 ml) drawn from healthy volunteers in an Ethylenediaminetetraacetic acid-containing tube at 2,000 rpm for 10 min at 20 °C. The RBC pellet was suspended in PBS at 10% volume by volume. Before use, the RBC suspension was diluted in PBS 1:10 proportion. Moreover, 100 ml aliquots from the suspension were mixed with 100 ml of AITC at a different concentration in the same buffer in Eppendorf tubes. Besides, 1% Triton X 100 was used for total hemolysis. After incubation for 1 h at 37 °C, it was centrifuged for 10 min at 2,000 rpm at 20 °C. Optical density was obtained at 450 nm after 150 μl of supernatant was transferred to a microtiter plate with a flat bottom. All the experiments were performed in triplicates [ 21 ]. The hemolysis percentage was calculated using the following formula:

% of Haemolysis = [[A_450_ of test compound treated Sample - A_450_ of buffer treated sample] / [A_450_ of 1%TritonX 100 treated sample - A_450_ of buffer treated sample]] × 100.

### Gene expression study with real-time polymerase chain reaction

To study the expressions of signal transduction genes involved in germ tube formation of *C. albicans*, RNA was extracted from *C. albicans* culture from germ tube formation. The 1×10^6^ cells were incubated in RPMI-1640 medium for 90 min at 37 °C with constant shaking in the presence and absence of AITC at its morphogenesis inhibitory concentration. The RNA was extracted with a RNeasy mini kit (QIAGEN, Valencia, CA, USA) and was reverse transcripted to cDNA using Super Script III (Invitrogen, Life technologies, Camarillo, CA, USA).

The PCR was carried out (Biorad Real-Time PCR Machine, 0.2 ml, 96 wells) in 96-well PCR plates with the help of UNI SYBR GREEN SUPERMIX. The quantitative PCR reaction total volume was 10 μl. Primers purchased from geneOmbiome Technologies Pvt Ltd.; Pune (Table 1) were added to SYBR GREEN SUPER MIX in a predetermined ratio. Expressions of the gene were analyzed with the help of a thermal cycler (Real Time System Bio-Rad Laboratories, Inc., Hercules, CA, USA) [ 13 ].

**Table 1:** Gene-specific primers used for Real-Time Polymerase Chain Reaction

Gene name	Primes	16783054762500Sequence (5’ → 3’)
Actin	ACTIN-F	5’ATGGACGGTGAAGAAGTTGC 3’
	ACTIN-R	5’ACCTCTTTTGGATTGGGCTTCA 3’
Ras-like protein 1	RAS1-F	5’GGCCATGAGAGAACAATATA 3’
	RAS1-R	5’GTCTTTCCATTTCTAAATCAC 3’
Phosphodiesterase 2	PDE 2-F	5’ACCACCACCACTACTACTAC 3’
	PDE 2-R	5’ AAAATGAGTTGTTCCTGTCC 3’
Bypass of CYclic-AMP requirement	BCY 1-F	5′ CCC AAGCTTATGTCTAATCCTCAACAGCA 3′
	BCY 1-R	5′ GGG CTGCAGTTAATGACCAGCAGTTGGGT 3′
Enhanced filamentous growth protein 1	EFG 1-F	5’ TATGCCCCAGCAAACAACTG 3’
	EFG 1-R	5’ TTGTTGTCCTGCTGTCTGTC3’
Transcription activator TEC1	TEC 1-F	5’ AGGTTCCCTGGTTTAAGTG 3’
	TEC 1-R	5’ ACTGGTATGTGTGGGTGAT 3’
Extent of cell elongation protein 1	ECE 1-F	5’-CCCTCAACTTGCTCCTTCACC-3’
	ECE 1-R	5’-GATCACTTGTGGGATGTTGGTAA-3’
Extracellular signal-regulated kinase 1	CEK 1-F	5’ AGCTATACAACGACCAATTAA 3’
	CEK 1-R	5’ CATTAGCTGA ATGCATAGCT 3’
Serine/threonine-protein kinase STE7 homolog	HST 7-F	5’ ACTCCAACATCCAATATAACA 3’
	HST 7-R	5’ TTGATTGACGTTCAATGAAGA 3’
Chlamydomonas photolyase homolog 1	CPH1-F	5’ATGCAACACTATTTATACCTC 3’
	CPH1-R	5’CGGATATTGTTGATGATGATA 3’
Cell-division-cycle 35	CDC35-F	5’TTCATCAGGGGTTATTTCAC 3’
	CDC35-R	5’CTCTATCAACCCGCCATTTC 3’
Hyphal wall protein 1	HWP1-F	5’TGGTGCTATTACTATTCCGG 3’
	HWP1-R	5’CAATAATAGCAGCACCGAAG 3’
Multicopy Inhibitor of GAL gene	MIG1-F	5’CTTCAACTAGCCTATATTCCGATGG 3’
	MIG1-R	5’CTTTCT GTAGGTACCAACAACTAC 3’
Neuregulin 1	NRG1-F	5’CACCTCACTTGCAACCCC 3’
	NRG1-R	5’GCCCTGGAGATGGTCTGA 3’
Transcriptional repressor TUP1	Tup1-F	5’ GAGGATCCCATGTATCCCCAACGCACCCAG 3’
	Tup1-R	5’GGCGACGCGTCGTTTTTTGGTCCATTTCCAAATTCTG 3’

### In vivo study in silkworm animal model

Department of Zoology at Shivaji University in Kolhapur, India provided third-instar silkworm larvae (*Bombyx mori*), which were fed mulberry leaves and kept alive until they reached the fifth instar. For the present study, only 1.9–2.2 g of silkworm larvae was selected. Cells of *C. albicans* were cultured in YPD broth overnight before being washed and re-dissolved in phosphate-buffered saline. An insulin syringe was used to inject 1×10^6^ cells into the hemolymph through the dorsal surface of a silkworm larva. 

The planktonic MIC_50_ concentration (0.125 mg/ml) of AITC was injected into the hemolymph to assess its anti-*C. albicans* effectiveness. Silkworms injected with FLC and *C. albicans* were considered the standard. Silkworm injected with DMSO. The mortality of silkworms was measured every 8 h for a duration of 48 h. Throughout the studies, silkworm larvae were maintained at 25°C, and survival was noted. It should be mentioned that experiments were carried out in triplicates [ 22 ]. Specification of groups used in *in vivo* silkworm animal model experiments is mentioned in Table 2.

**Table 2:** Specification of groups used in *in vivo* silkworm animal model experiment

**Group I**	Positive control	Silkworm injected with *Candida albicans*
**Group II**	Negative control	Silkworm injected with PBS
**Group III**	Test	Silkworm injected with *C. albicans* + planktonic MIC_50_ concentration (0.125 mg/ml) of AITC drug
**Group IV**	DMSO	Silkworm injected with DMSO (1%)
**Group V**	Standard	Silkworm injected with *C. albicans* and fluconazole MIC_50_ concentration (0.15µg/ml)

PBS: Phosphate Buffer Saline, MIC_50_: Minimum Inhibitory Concentrations, AITC: Allyl Isothiocyanate, DMSO: Dimethyl Sulfoxide

### Statistical analysis

All experiments were carried out in triplicates and the mentioned values were the mean values obtained from three different observations. Values in the control and treatment groups for various molecules were compared using Student’s t-test. The *P*-values of < 0.05 were considered statistically significant. The *in vivo* experimental data was analyzed using the GraphPad Prism software (version 6.0, San Diego, CA).

### Ethical Consideration

The University Ethics Committee registration number is ECR/738/Inst/MH/2015/RR-21.

## Results

### Antifungal activity of AITC on C. albicans planktonic growth

**Figure 1:**
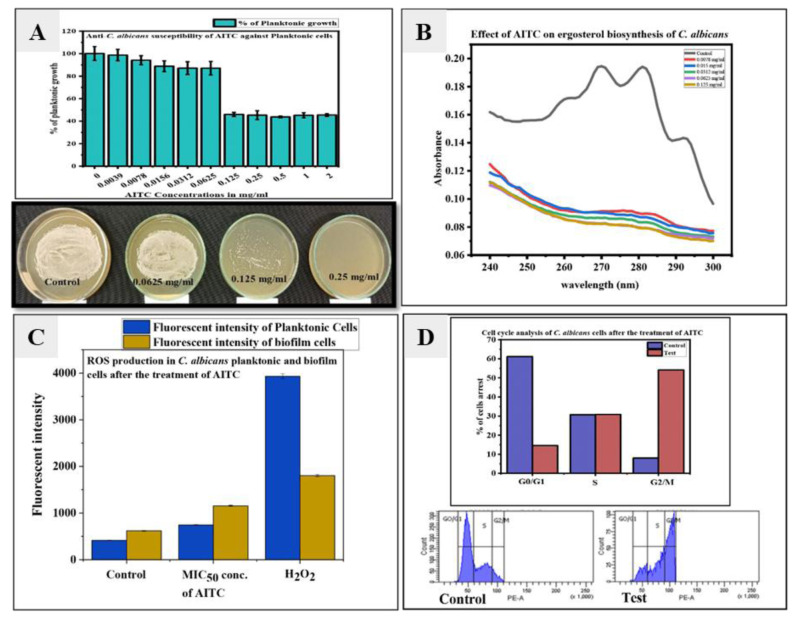
A. Planktonic growth inhibitory activity fungicidal nature of allyl isothiocyanate against *Candida albicans*. B. The effect of allyl isothiocyanate on ergosterol biosynthesis. C. Detection of ROS level after treatment of allyl isothiocyanate using DCFDA staining involved in the planktonic and biofilm cells of *C. albicans*. D. Flow cytometry analysis of cell cycle arrest in *C. albicans* planktonic cells with propidium iodide staining after allyl isothiocyanate treatment.

The inhibitory effect on planktonic growth of *C. albicans* was assessed by AITC (Figure 1A). The MIC_50_ of AITC for *C. albicans* was found to be 0.125 mg/ml (Figure 1A). The MFC of AITC was assessed by spread plate technique on a YPD plate with the help of MIC_50_ concentration and the results indicated that AITC was fungicidal in nature at its MIC_50_ and higher concentrations (Figure 1A). Fluconazole inhibits planktonic growth of *C. albicans* at 1 µg/ml concentration.

### Effect of AITC on C. albicans cell membrane

The cell membrane is the primary target for many standard antifungal drugs, like azoles and polyenes. Therefore, in the present study, we estimated total ergosterol content in *C. albicans* cells upon treatment with AITC. The impact of AITC on the cell membrane of *C. albicans* was examined using ergosterol biosynthesis. Ergosterol concentrations of AITC-treated and untreated *C. albicans* cell membranes were measured. Untreated control cells show characteristic peaks that signify ergosterol production. However, in this study, ergosterol biosynthesis suppression in *C. albicans* cells treated with AITC at planktonic MIC_50_ concentration and below MIC_50_ concentration in the range of 0.0078 to 0.125 mg/ml was represented by a flat curve (Figure 1B).

### Allyl isothiocyanate induced intracellular reactive oxygen species generation in C. albicans

Under extremely stressful conditions, *C. albicans* are known to produce ROS. High concentrations of ROS, including superoxide anion radicals, hydroxyl radicals, hydrogen peroxide, hypochlorous acid, and hydroperoxyl radicals, may interact with biological components, such as lipids, proteins, and nucleic acids, resulting in oxidative stress and ultimately cell death [ 23 ]. 

The fluorescent dye 2',7'-dichlorodihydrofluorescein diacetate was used to assess AITCs capacity to stimulate endogenous ROS generation in *C. albicans* (Figure 1C). Figure 1C shows that AITC increased fluorescence intensity, which demonstrates the capacity of AITC to boost endogenous ROS production. The AITC promoted intracellular ROS generation at its 0.125 mg/ml planktonic inhibitory concentration. However, an increase in the production of ROS after the treatment of AITC, compared to non-treated *C. albicans* cells may be responsible for the eradication of *C. albicans* planktonic growth. Along with this, AITC treatment also increased ROS production in *C. albicans* biofilm at 0.5 mg/ml concentration. An increase in ROS production in biofilm cells of *C. albicans* may be a reason for the anti-biofilm activity of *C. albicans*.

### Effect of allyl isothiocyanate on cell cycle

We examined the impact of AITC on the *C. albicans* cell cycle. In order to understand how AITC affected DNA replication and cell division, two crucial and tightly controlled processes for the growth and multiplication of a cell, cell cycle study was conducted. Any irregularity in DNA replication and cell division triggers the DNA damage checkpoint pathway, which halts all processes related to growth and division until the damage is repaired. 

In a cell cycle analysis investigation, *C. albicans* cells were exposed to a planktonic inhibitory dose of AITC (0.125 mg/ml) and compared to untreated cells. The DNA content present during various cell cycle stages was measured based on the fluorescence intensity produced by propidium iodide to confirm cell cycle arrest in *C. albicans*. Regarding the control cells, 61.1% of them were arrested in G0/G1 phase, 30.7% in the S phase, and 8.0% in the G2/M phase while regarding the test cells, 14.6% cells were arrested in G0/G1 phase, 30.9% in S phase and 54. % in G2/M phase (Figure 1D). The results suggested that AITC arrested cells in the G2/M phase of *C. albicans*. 

### Inhibitory effect of allyl isothiocyanate on germ tube formation of Candida albicans

**Figure 2:**
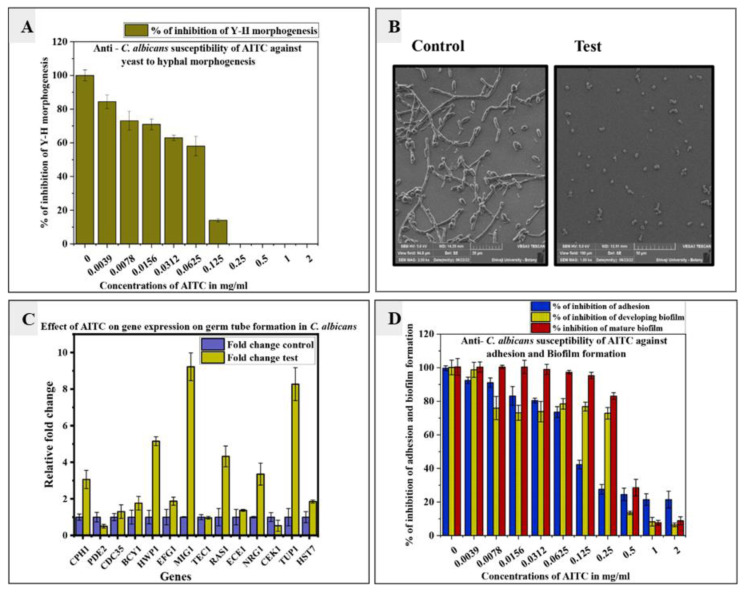
A. Inhibitory effects of allyl isothiocyanate on *Candida albicans* germ tube formation in a concentration-dependent manner. B. Scanning electron microscopy analysis to assess the effect of 0.125 mg/ml of allyl isothiocyanate on germ tube formation of *Candida albicans*. C. Transcriptional profiles of *Candida albicans* cells on germ tube formation treated with and without allyl isothiocyanate. D. Effect of allyl isothiocyanate on the adhesion, development of biofilm, and mature biofilm of *Candida albicans* to polystyrene plates.

In this study, the effect of AITC on germ tube formation of *C. albicans* was analyzed using FBS growth media. The AITC inhibited the germ tube formation in a concentration-dependent manner. The effect was studied at various concentrations ranging from 0.0039 to 2 mg/ml. At 0.125 mg/ml concentration, AITC completely inhibited the germ tube formation (Figure 2A). In the current study, FLC did not inhibit germ tube formation. 

### Morphology analysis of Candida albicans germ tube on exposure to allyl isothiocyanate

The morphological analysis of *C. albicans* cells was further assessed for germ tube inhibition by SEM. *Candida albicans* control cells consisted of germ tube, whereas cells treated with 0.125 mg/ml concentration of AITC showed that germ tube formation was completely inhibited (Figure 2B).

### Effect of allyl isothiocyanate on signal transduction gene expression involved in germ tube inhibition of Candida albicans

By usage of Qrt-PCR analysis, the impact of AITC on *C. albicans* germ tube inhibition was evaluated at the transcriptional level. The impact of 0.125 mg/ml concentration of AITC on the germ tube revealed a notable decrease in *C. albicans* hyphal development, compared to the non-treated control. Genes involved in the germ tube formation of *C. albicans* have their expression changed by the AITC treatment. The qRT-PCR analysis showed a relative fold change in the gene expressions. Allyl isothiocyanate inhibited virulence factors, like germ tube formation by downregulation of *PDE2*, *CEK1*, and *TEC1* by 2.54, 1.91, and 1.04-fold change, respectively, and upregulation of *MIG1*, *NRG1*, and *TUP1* by 9.22, 3.35, and 7.80-fold change, respectively (Table 3, Figure 2C). 

**Table 3:** Relative fold changes in the gene expressions involved in the signal transduction pathway of *Candida albicans* after the treatment of AITC

Genes	Fold change (FC)
*CPH1*	Upregulated (2.43-fold)
*PDE2*	Downregulated (2.54-fold)
*CDC35*	Upregulated (1.29-fold)
*BCY1*	Upregulated (1.69-fold)
*HWP1*	Upregulated (3.99-fold)
*EFG1*	Upregulated (1.78-fold)
*MIG1*	Upregulated (9.22-fold)
*TEC1*	Downregulate (1.04-fold)
*RAS1*	Upregulated (4.08-fold)
*ECE1*	Upregulated (1.29-fold)
*NRG1*	Upregulated (3.35-fold)
*CEK1*	Downregulated (1.91-fold)
*TUP1*	Upregulated (7.80-fold)
*HST7*	Upregulated (1.80-fold)

### Effect of allyl isothiocyanate on adhesion of Candida albicans

Adhesion plays a vital role in biofilm formation and infection of *C. albicans*. The inhibitory effect of AITC and FLC on *C. albicans* on a polystyrene surface was quantified by XTT metabolic assay. The AITC inhibited the adhesion to the polystyrene surface to an extent of 50% at a concentration of 0.125 mg/ml. Moreover, AITC concentrations of 0.25 mg/ml, 0.5 mg/ml, 1 mg/ml, and 2 mg/ml significantly decreased adhesion to an extent of 28%, 25%, 21% and 21%, respectively (Figure 2D) while FLC was unable to inhibit the adhesion of *C. albicans* cells.

### Effect of allyl isothiocyanate on biofilm formation (developing and mature biofilm)

The AITCs anti-biofilm activity was evaluated against the *C. albicans* ATCC 90028 strain. At a dose of 0.5 mg/ml, AITC suppressed the early or emerging biofilm and at the same concentration, AITC suppressed the mature biofilm as shown by the XTT metabolic assay (Figure 2D).

### Toxicity effect of allyl isothiocyanate on human red blood cells

**Figure 3:**
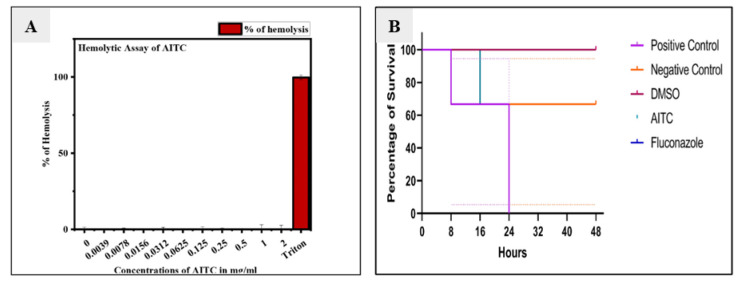
A. Effect of allyl isothiocyanate on human red blood cells. B. Effects of allyl isothiocyanate on *Candida albicans* infected silkworm. The graph indicates the percentage of worm survival after exposure of *C. albicans* to allyl isothiocyanate for 48 h.

Toxicity of AITC was analyzed by *in vitro* hemolytic activity on human RBCs. It was observed that AITC was non-hemolytic in nature in a concentration range of 0.0039-2 mg/ml (Figure 3A). 

### In vivo study using silkworm animal model

*In vivo* experiment was conducted on silkworm (*Bombyx mori*) to examine the antifungal efficacy of AITC against *C. albicans*. Silkworms injected with *C. albicans* cells were considered positive control and silkworms injected only with PBS were considered negative control. Silkworms injected with AITC (0.125mg/ml) and *C. albicans* were considered the test group. Positive control silkworms died within 24 h, while negative control silkworms completed their life cycle and underwent the cocoon phase. However, silkworms injected with AITC and *C. albicans* cells survived and completed their life cycle (Figure 3B).

## Discussion

Isothiocyanates are well-known antimicrobial substances used to fight bacteria and potentially harmful fungi. It is surprising that little research has been performed on how ITCs affect yeasts, like *C. albicans*. Very few studies have shown the effect of isothiocyanate on *C. albicans*. However, Pereira et al. in 2020 [ 24 ] reported that benzyl isothiocyanate (BITC) inhibits germ tube formation in *C. albicans*. They demonstrated that BITC treatment increased cell size and oxidative stress within the cell and significantly altered the ultrastructure of the cell wall. The BITC treatment causes adverse effects on the inner layer of the cell wall by interfering with the formation of glucans or the structure of the cell wall in *C. albicans*. The yeast-to-hyphal transition is a significant pathogenic factor in *C. albicans* infections. This inhibition is most likely caused by the effect on the cell wall of *C. albicans*. Overall, these effects may be able to influence *C. albicans* colonization by limiting or restraining the invasiveness of the organism and therefore, allowing host defenses to respond [ 24 ].

Anti-*Candida* activity of AITC alone and in combination with FLC was explored by Raut et al. in 2017 [ 12 ]. The activity of AITC against *C. albicans* pathogenicity and planktonic growth was concentration-dependent. At 1 mg/ml, the biofilm was significantly (P≤0.05) inhibited by AITC. Notably, the biofilm was not formed when 0.004 mg/ml of FLC and 0.125 mg/ml of AITC were combined. The AITC-FLC combination also significantly (P≤0.05) suppressed the developed biofilms. The fractional inhibitory concentration indices, which ranged from 0.132 to 0.312, showed that AITC and FLC worked together to prevent the development of both early and mature biofilms. Toxicity study analysis has suggested that AITC alone and in combination with FLC causes no hemolysis [ 12 ]. 

The present study brings new insight into the mechanism of action of AITC against *C. albicans*. Ergosterol is an important component of fungal cell membranes and a prime target of antifungal agents. The AITC inhibited ergosterol biosynthesis at its MIC and sub-MIC concentrations (Figure 1B). Both human and fungal cells are eukaryotic, and since antifungal medications target both of these cell types, there are fewer accessible targets for pharmacological action and significant adverse effects for patients. Many newer antifungals have been identified by appropriate studies. However, these molecules have not yet reached the clinical application levels. 

Available antifungal drugs belong to various groups, such as azoles (FLC, itraconazole, ketoconazole, miconazole, and clotrimazole), polyenes (Amphotericin B, Nystatin), Allylamines, Thiocarbamates, Morpholines, 5-fluorocytosine (an analog of deoxyribonucleic acid), and Echinocandins. Azoles mainly act via targeting ergosterol biosynthesis. In the endoplasmic reticulum of the fungal cell, azoles prevent the enzyme lanosterol 14-demethylase from converting lanosterol into ergosterol, a component that is essential for the building of the plasma membrane structure of the fungus. As a result, the hazardous substances 14-methyl-3, and 6-diol will build up. As ergosterol concentration decreases, it causes changes to the cell membrane structure that prevents *C. albicans* growth [ 25 , 26 ]. In the current study, it was found that AITC significantly inhibited ergosterol biosynthesis in *C. albicans* (Figure 1B).

In the current study, it was found that AITC increased the ROS production in both planktonic and biofilm cells of *C. albicans*, compared to the control (Figure 1C). According to the reports, substances responsible for induction of ROS production may have promising antifungal properties. Many studies have shown that ROS-induced *C. albicans* apoptosis occurs in the presence of acetic acid, resveratrol, farnesol, and antimicrobial peptides. The ROS has apoptotic effects on a variety of cell types, including *C. albicans*. The fungicidal nature of commonly used antifungal medications, such as azoles, has been linked to their increased ROS effects in addition to their target-specific actions. Additionally, miconazole-tolerant Candida cells have higher activity in inactivating ROS. Superoxide dismutase is a crucial component of *C. albicans* pathogenicity and one of the enzymatic and non-enzymatic antioxidant defense mechanisms found in *C. albicans* [ 27 ]. Therefore, the planktonic growth and virulence factor inhibiting the ability of AITC might be due to ROS production.

In addition, this study demonstrated that AITC triggered the *C. albicans* cell cycle by arresting cells at the G2/M phase (Figure 1D). In higher eukaryotes, the relationship between cell cycle regulation and the induction of apoptosis is still unknown. According to reports, pro-apoptotic therapies cause the cell cycle of *C. albicans* to arrest in the G2/M phase. Wani et al. in 2021 and Phillips et al. in 2003 [ 28 , 29 ] reported that the DNA damage repair checkpoint and the G2/M phase coincide. Induction of cellular death in yeast cells may cause DNA breakage by the production of ROS which, in turn, causes G2/M cell cycle arrest [ 28 , 29 ]. 

SEM was used to examine how AITC affected the morphology of the *C. albicans*. The SEM analysis revealed that germ tube induction was visible in the control sample (Figure 2B), while it was completely absent in the AITC-treated sample and only smooth-walled spherical entities were present. Germ tube formation is a crucial component of the pathogenicity of *Candida*. In biofilms, the hyphae help the structure to become stable. To enhance antifungal therapy, inhibition of germ tube formation plays a crucial role [ 6 , 30 ].

We have investigated the expression of genes that are involved in the signal transduction pathway of germ tube formation. RAS1-cAMP-PKA and CEK1-Mitogen Activated Protein Kinase (MAPK) are two components of the germ tube formation signal transduction pathway [ 31 ]. The AITC targets a crucial element in the germ tube formation pathway. The CPH1 and EFG1 are the major transcription regulators of filamentous growth [ 5 ] which were upregulated by 1.43-fold and 1.78-fold, respectively (Figure 2C). *MIG1*, *NRG1*, and *TUP1* expressions were upregulated by AITC 9.22, 3.35, and 7.80-fold change respectively. The MIG1, TUP1, and NRG1 play crucial roles in the suppression of the Y-H form transition as they are negative regulators [ 32 ]. 

The expression of *PDE2*, *TEC1* and *CEK1* genes underwent 2.54-, 1.04-, and 1.91-fold decreases, respectively (Figure 2C). The PDE2, a high-affinity phosphodiesterase, was necessary for *C. albicans* hyphal growth and cell wall integrity. The downregulation of *PDE2* elevates cAMP levels, prevents normal hyphal development in a hypha-inducing liquid medium, and inhibits the formation of biofilms [ 33 ]. A transcription factor TEC1 is connected to morphogenesis and functions in controlling hyphal differentiation [ 34 ]. In *C. albicans*, four MAPK have been discovered, which play a significant role in the development of cell walls and biofilms. One of these four, the CEK1-MAPK, plays a key role in filamentous growth and is a key determinant of virulence in *C. albicans*. Downregulation of the *CEK1* gene expression can cause a reduction in germ tube or hyphal production and diminish virulence in *C. albicans* [ 35 ].

In order to stop the global spread of infections that are multi-drug resistant, there is an increasing need for new antibiotics. Silkworms are susceptible to the same pathogenic bacteria and fungi that may infect humans, and the same drugs that are used to treat human infections can also cure infected silkworms. The simplicity, low cost, and lack of ethical concerns of the silkworm as an animal model are some of its distinguishing features. The silkworm infection model is a good choice for evaluation of the therapeutic efficacy of antimicrobial medicines as it has a conserved gene sequence and similar pharmacokinetics to mammals [ 36 , 37 ]. 

In many studies it was demonstrated that the dose (ED50) needed to treat 50% of fatal infections in silkworms is comparable to that needed to treat 50% of fatal infections in mice, indicating that the pharmacokinetics of these antibiotics are similar in silkworms and mammals. In many bacterial infection studies, silkworm has been used as a model to test the therapeutic potency of different antibacterial compounds. 

Researchers have been able to successfully identify lysocin E, a novel antibiotic with a mode of action that involves binding to menaquinone to cause membrane damage and bactericidal activity. Other therapeutically effective novel antibiotics, including nosokomycin and ASP2397 (VL-2397), were discovered as a result of the same method used to screen *Candidate* antibiotics. This suggests that the silkworm antibiotic screening strategy is quite successful in identifying new antibiotics [ 36 , 37 ]. Similarly, in the current study, silkworm was used as a model organism to screen the drug. Based on the *in vivo* study, it was concluded that Planktonic MIC_50_ concentration (0.125 mg/ml) of AITC inhibited *C. albicans* infection and did not cause toxicity to the silkworm (Figure 3B). The findings of this study suggested that AITC may be a promising molecule for the development of a future antifungal drug.

## Conclusion

The AITC is a potential inhibitor of growth and virulence factors in *C. albicans*. It alters the sterol profile and blocks ergosterol biosynthesis. Moreover, AITC produces ROS in both planktonic and biofilm cells and arrests cells at the G2/M pre-apoptic phase. The AITC alters the expression of genes involved in the signal transduction pathway which inhibits germ tube formation by downregulating *PDE2*, *CEK1*, and *TEC1* and upregulating *TUP1*, *MIG1*, and *NRG1* genes. Toxicity assay has revealed that AITC can be used as an alternate therapeutic option to treat candidiasis as it is non-toxic to human RBCs. *In vivo* study has proved that AITC also increases the survival rate of silkworms by inhibiting *C. albicans* infection. There is a need for further evaluation of AITC by performing *in vivo* studies on mice.
